# Supplementary material for: Expression profiling and in situ screening of circular RNAs in human tissues
Source: Sci Rep. 2018 Nov 16;8:16953. doi: 10.1038/s41598-018-35001-6 (PMC6240052; doi:10.1038/s41598-018-35001-6)
Supplement: Supplementary file 1 — Supplementary Figures [file 41598_2018_35001_MOESM1_ESM.pdf]

Supplementary figures

Expression profiling and in situ screening of circular RNAs in human tissues

Ammar Zaghlool[1], Adam Ameur[1], Chenglin Wu[2], Jakub Orzechowski Westholm[2], Adnan Niazi[1], Manimozhi Manivannan[3], Kelli Bramlett[3], Mats Nilsson[1,2], Lars Feuk[1*]

[1] Department of Immunology, Genetics and Pathology, Science for Life Laboratory, Uppsala University, Uppsala, Sweden

[2] Science for Life Laboratory, Department of Biochemistry and Biophysics, Stockholm University, Stockholm, Sweden

[3] Clinical Sequencing Division, Life Science Solutions Group, Thermo Fisher Scientific, San Francisco, CA, USA

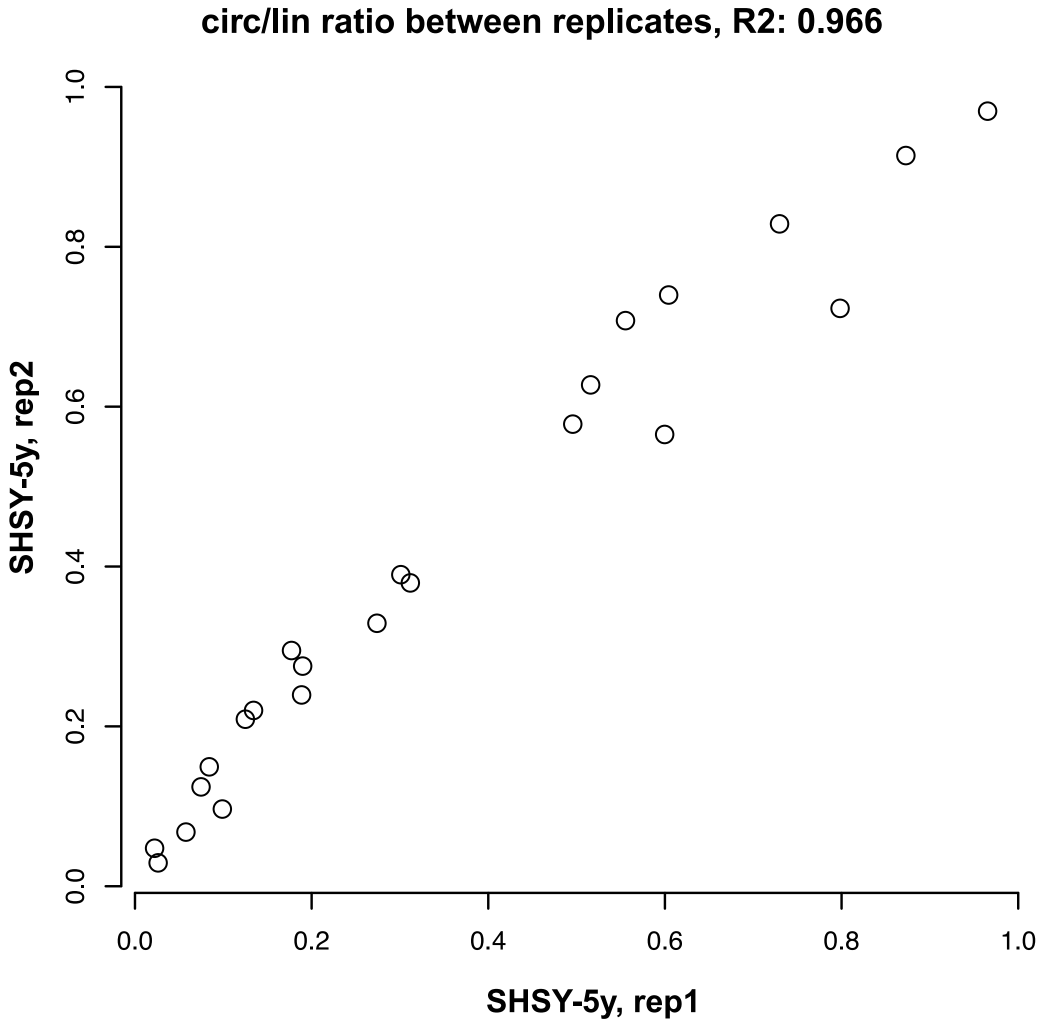

Supplementary figure S1

Correlation of circRNA/linear RNA ratios (raw read counts) obtained from the circRNA AmpliSeq panel, for two replicate RNA samples from the SHSY-5Y cell line.

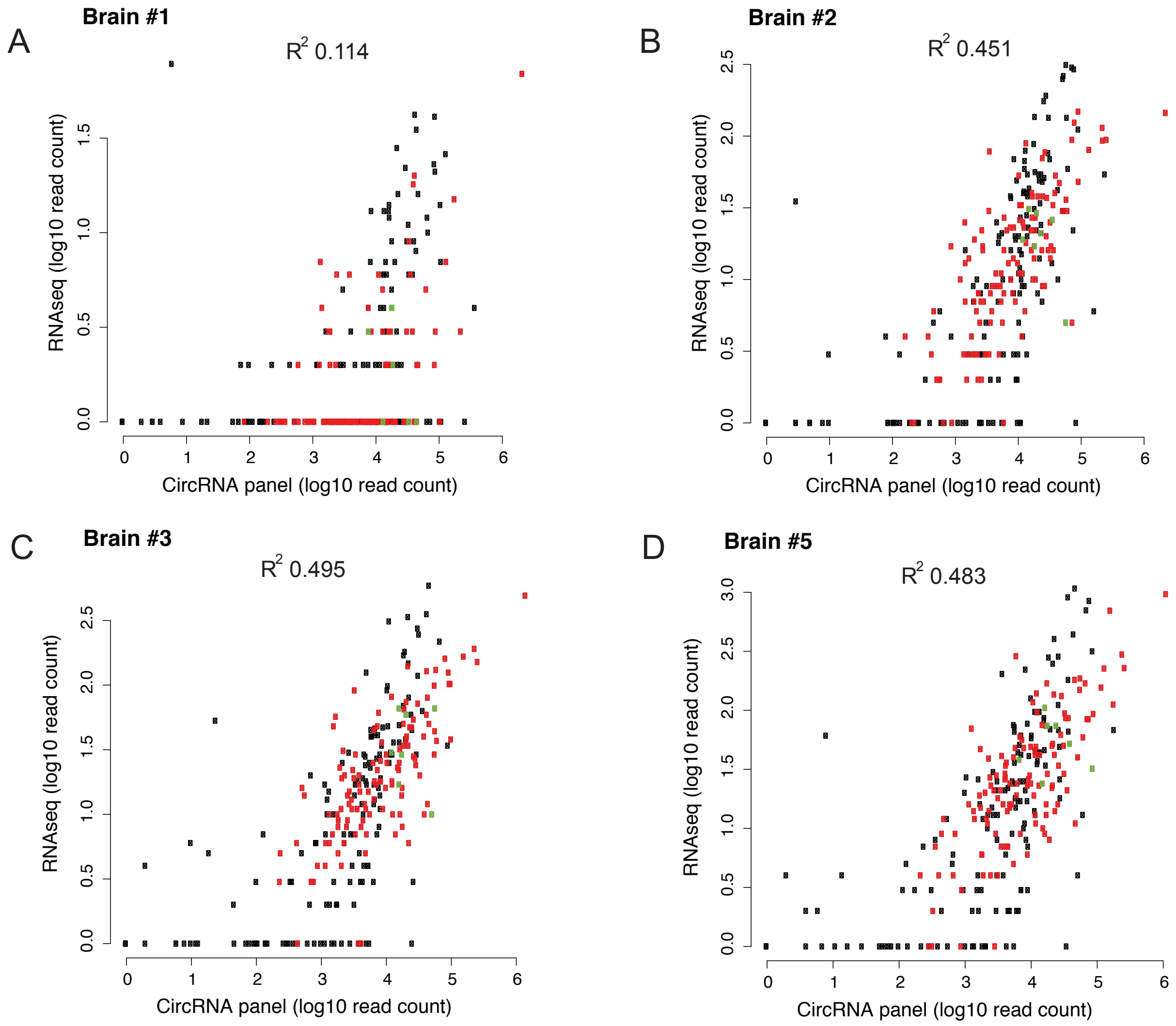

Supplementary figure S2

Quantification of circRNA expression levels from total RNA-seq (y-axis) and the AmpliSeq circRNA panel (x-axis) in brain samples. The axis scales are logarithmic and the value 1 was added to each target, implying that non-expressed have a value of 0 in the plot. Each dot represents a target amplicon in the circRNA panel, either a circular RNA (red) or a linear RNA (black).

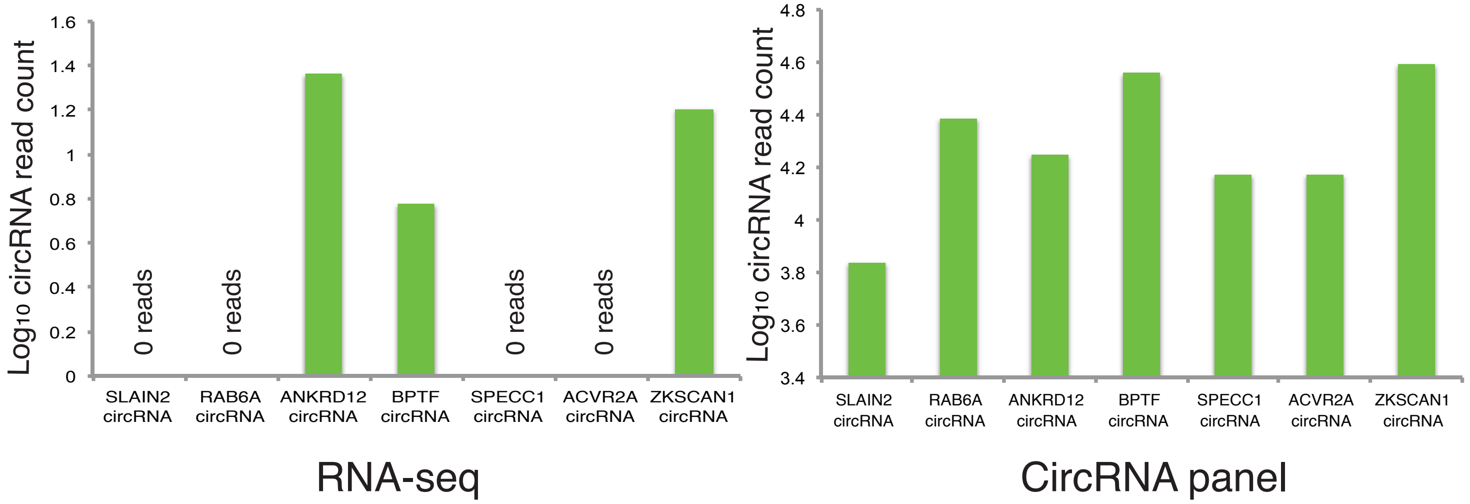

Supplementary figure S3

Log 10 of the read counts for circRNA analyzed in Figure 2D for RNA-seq data and the CircRNA panel

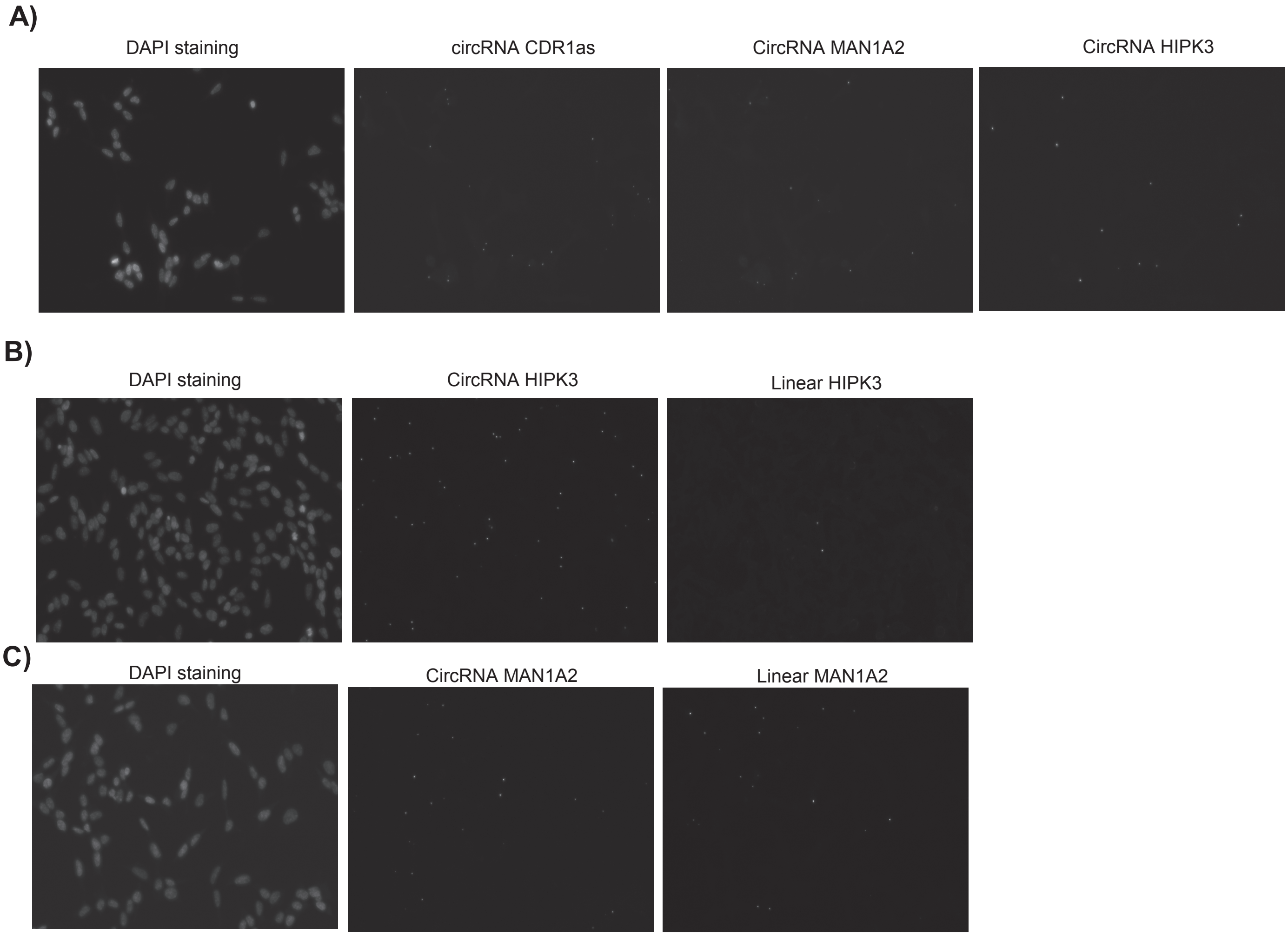

Supplementary Figure S4

Raw data for circRNA and linear RNA detectiom in SHSY-5y cells. A) Gray scale images for figure 5A. B) Gray scale images for the detection of circRNA and linear HIPK3 transcripts for figure 5C. C) Gray scale images for the detection of circRNA and linear MAN1A2 transcripts for figure 5C.

A

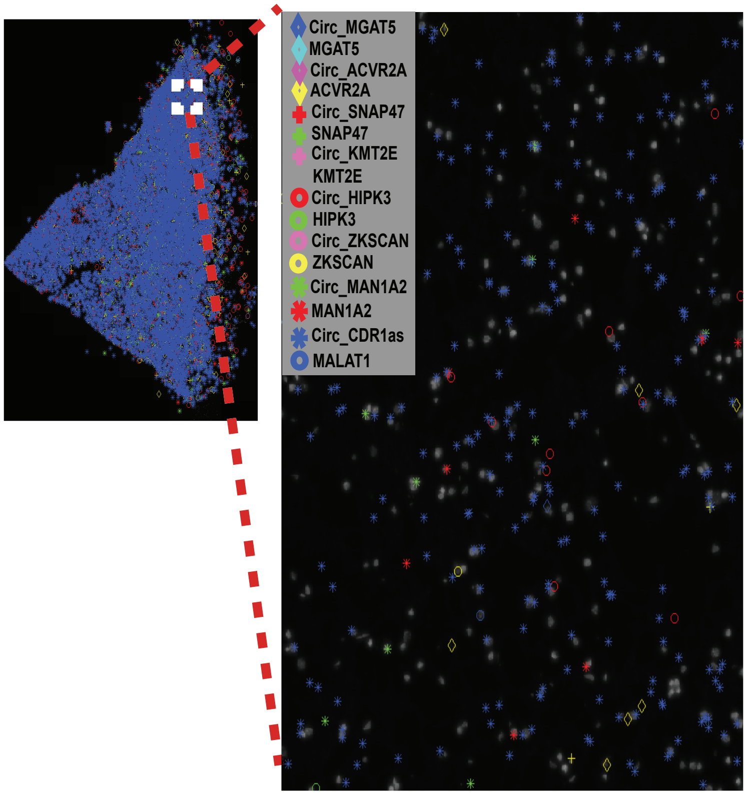

B

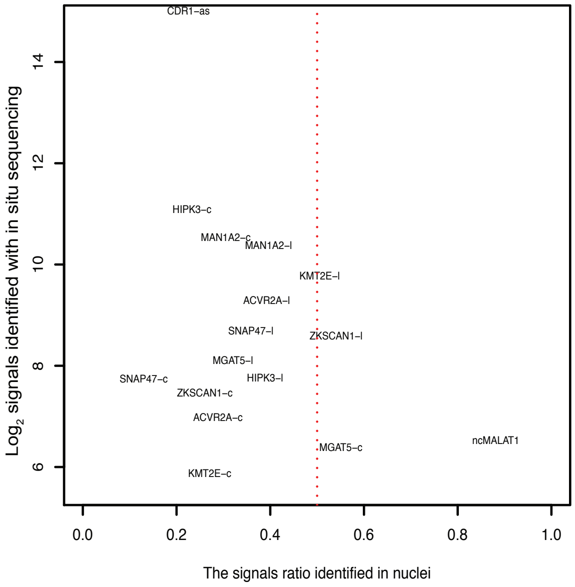

Supplementary figure S5
Same as figure 6 but with brain sample 4
